# Gender Differences in Misdiagnosis and Delayed Diagnosis among Adults with Autism Spectrum Disorder with No Language or Intellectual Disability

**DOI:** 10.3390/brainsci11070912

**Published:** 2021-07-09

**Authors:** Camilla Gesi, Giovanni Migliarese, Sara Torriero, Martina Capellazzi, Anna Caterina Omboni, Giancarlo Cerveri, Claudio Mencacci

**Affiliations:** 1 Department of Psychiatry and Addiction, ASST Fatebenefratelli-Sacco, 20157 Milan, Italy; gesi.camilla@asst-fbf-sacco.it (C.G.); s.torriero@libero.it (S.T.); anna.omboni@asst-fbf-sacco.it (A.C.O.); claudio.mencacci@asst-fbf-sacco.it (C.M.); 2 Department of Psychiatry and Addiction, ASST Pavia, 27100 Pavia, Italy; 3 School of Medicine and Surgery, University of Milano—Bicocca, 20126 Milan, Italy; martina.capellazzi@gmail.com; 4 Department of Psychiatry and Addiction, ASST Lodi, 26900 Lodi, Italy; giancarlo.cerveri@asst-lodi.it

**Keywords:** Autism Spectrum Disorder, gender, female phenotype, misdiagnosis, late diagnosis, Autism Spectrum Domains, sensory reactivity, comorbidity

## Abstract

Autism Spectrum Disorder (ASD) is often unrecognized, especially in mild forms and in women. Studies evaluating features associated with missed/misdiagnosis in men and women with ASD are warranted. 61 subjects (22 females, 39 males, age 28.5 ± 10.8 years) with ASD with no language/intellectual deficit were enrolled in the service for the treatment of psychiatric comorbidities in adults with ASD of the ASST Fatebenefratelli-Sacco in Milan (Italy). A detailed clinical history was gathered, and two self-report questionnaires (Autism Spectrum Quotient-AQ and Adult Autism Subthreshold Spectrum-AdAS Spectrum) were administered. 75.4% received their ASD diagnosis average eight years later than the first evaluation by mental health services. Compared to males, females showed a significantly greater delay in referral to mental health services and a significantly higher age at diagnosis of ASD. Among men, diagnostic delay inversely correlated with scores on the AdAS Spectrum total, Verbal communication, Empathy and Inflexibility and adherence to routine domains. Among women, diagnostic delay positively correlated with the Attention to detail score while the age at diagnosis of ASD positively correlated with the AdAS Spectrum Verbal communication and Restricted interests and rumination domain scores. Females were less likely to be correctly diagnosed and more likely to be misdiagnosed at first evaluation than men. Females reported significantly higher scores than men in the Hyper/Hyporeactivity to sensory input domain only among subjects who were misdiagnosed. Our findings provide gender-specific information about ASD patients seeking help for comorbid conditions and might be a primary ground for future research.

## 1. Introduction

Autism Spectrum Disorder (ASD) is a neurodevelopmental condition characterized by persistent deficits in social communication and interaction and a pattern of restricted interests and/or repetitive behaviors [1]. Despite a large body of research highlighting the clinical and neurobiological continuity among different forms of autism, leading to their subsumption into one single spectrum, it has been pointed out that ASD includes subjects with a broad variability in symptoms, associated features, and degrees of impairment [2]. Some authors especially questioned the appropriateness of ASD criteria for detecting mild forms of autism, with no language or intellectual disability, and featuring a high level of functioning, formerly referring to the categories of high-functioning autism and Asperger Disorder [3]. Criticisms also pointed out the high frequency of missed ASD diagnoses not only in late- or nonreferred subjects, but also among those who sought an early evaluation [4].

The clinical heterogeneity of ASD is at least partially due to the high comorbidity with other mental disorders, especially when represented in high-functioning forms. While the comorbidity with other psychiatric conditions might be over-represented among patients with mild ASD compared to those with severe forms [5], lifetime estimates of overlapping disorders in this subgroup are quite large, ranging from 70 to about 80% [6,7]. It has been suggested that ASD might both share a common etiopathological root with other disorders and be itself the ground where other disorders flourish [8,9]. The role of ASD as a psychopathological risk factor is confirmed by a number of studies, especially when focusing on the high level of distress in everyday life due to the lack of interpersonal and communication skills and leading to anxiety, depression, trauma/stress-related symptoms and suicidal thoughts [10,11,12,13,14]. This might be especially critical for mild forms of ASD, which often remain unknown through childhood and make their first appearance during adolescence or adulthood, when social demands exceed the individual coping ability [1]. In this subgroup of patients, first referral to mental health services may be prompted late in time by the abrupt onset of new psychiatric symptoms rather than by mild and long-standing autistic-related difficulties [15,16,17].

ASD diagnosis is often challenged by the presence of coexisting disorders, as multiple conditions may overshadow or shape one another, giving rise to atypical phenotypes and hampering diagnostic reasoning [18,19,20]. Alongside the risk of missing an ASD diagnosis, a steadily increasing number of studies has brought attention to the high chance of patients with mild ASD also being misdiagnosed with a variety of conditions due to the phenotypic overlap among multiple disorders [21]. These misdiagnoses stem, in part, from a widespread unfamiliarity with the features of ASD in adults, preventing clinicians from disentangling complex clinical pictures and, even more importantly, from providing appropriate treatment.

The issue of missed and misdiagnoses among ASD subjects might be especially pronounced among females [1,20]. Several studies highlighted the more complex presentation of ASD in women [22] and the greater likelihood of females hiding their autistic symptoms, leading to camouflage among a neurotypical population [23] and hindering the diagnostic process [20,24]. Previous studies have shown that women with ASD tend to report lower scores in questionnaires assessing some autistic dimensions [25,26] and that certain mental disorders, such as anorexia, borderline personality disorder and social phobia, often stem from autism-related underpinnings, lagging unrecognized behind comorbid disorders [27]. Since extant data are still insufficient to draw gender-specific phenotypes among ASD populations, instruments specially tailored to unmask autism among women and studies providing information about gender-specific clinical features are keenly warranted in order to reduce missed and misdiagnoses among ASD sufferers and provide appropriate treatment for both ASD and concurrent conditions.

Based on the above premises, the present observational, cross-sectional study has been carried out in service of the treatment of psychiatric comorbidities in adults with a principal diagnosis of ASD, with the following aims: 1. assess autism spectrum symptoms among referred males and females; 2. evaluate sex differences with regard to diagnostic delay and the rate of missed and misdiagnosis; 3. test whether specific domains of ASD symptomatology are associated with a greater diagnostic delay or higher likelihood of misdiagnosis among ASD women and males respectively.

## 2. Materials and Methods

### 2.1. Participants

The study sample included sixty-three adult patients consecutively referred to the outpatient service for adults with ASD of the ASST Fatebenefratelli-Sacco in Milan (Italy), a tertiary center for the treatment of psychiatric comorbidities in adults with a principal diagnosis of ASD without language or intellectual disability. Subjects were enrolled between May and December 2020, based on the following criteria: (1) a clinical diagnosis of ASD, with no language or intellectual disability, (2) subjects 18 years old and above, (3) the ability to provide informed consent for participation in the study. Principal and comorbid diagnoses were based on Diagnostic and Statistical Manual of Mental Disorders (DSM-5) criteria through an accurate clinical investigation, performed by psychiatrists with expertise in ASD. The study was conducted in accordance with the Declaration of Helsinki, and all participants gave written informed consent to take part in the study. As a relevant institutional review board for low-risk studies, the Department of ASST Fatebenefratelli-Sacco of Milan approved the study protocol.

### 2.2. Materials

Clinical and sociodemographic data were gathered during the first evaluation on a specially prepared form, using as many sources of information as available (patients’ and relatives’ recall, written clinical reports, etc.). In addition, all participants were asked to fill out two self-administered questionnaires, to obtain a detailed assessment of ASD symptoms. The Autism-Spectrum Quotient (AQ) is a widely used questionnaire, developed about fifteen years ago, providing a self-report measure of autistic traits for use in adults with a normal IQ [28]. It comprises 50 questions, assessing five different areas: social skill, attention switching, attention to detail, communication, and imagination. The AQ has been used as a screening tool for ASD in the general population, as well as to evaluate autistic symptoms within ASD populations and other clinical groups [29,30]. The AdAS Spectrum was recently developed to provide a granular assessment of autism spectrum symptoms across the lifetime of an individual [31]. It allows one to assess a wide array of clinical and nonclinical manifestations of ASD, with specific attention devoted to some gender-specific features and to sensory reactivity, recently included as a criterion symptom in the DSM-5. The questionnaire includes 160 items exploring the wide spectrum of manifestation of autism, organized into seven domains: Infancy/adolescence, Verbal communication, Non-verbal communication, Empathy, Adherence to routine and inflexibility, Restricted interests and rumination, Hyper-/hyporeactivity to sensory input. The instrument showed a high internal consistency, sound test–retest reliability, and strong convergent validity with alternative dimensional measures of autism, and it has already been used in a number of studies aiming to assess ASD symptoms and autistic traits in various populations [32,33].

### 2.3. Statistical Analysis

The demographic and clinical characteristics of males and females with ASD were compared by means of the Chi-square test for categorical variables and the Student T-test for continuous variables. Correlation analyses were used to investigate which symptom dimensions were associated with delayed diagnosis of ASD among women and men respectively. The effects of gender, misdiagnosis and of their possible interaction on AdAS Spectrum domain scores were analyzed by means of seven two-way ANOVA analyses. Statistics were conducted using SPSS, version 26 [34].

## 3. Results

### 3.1. Description of the Study Sample

**Figure 1:**
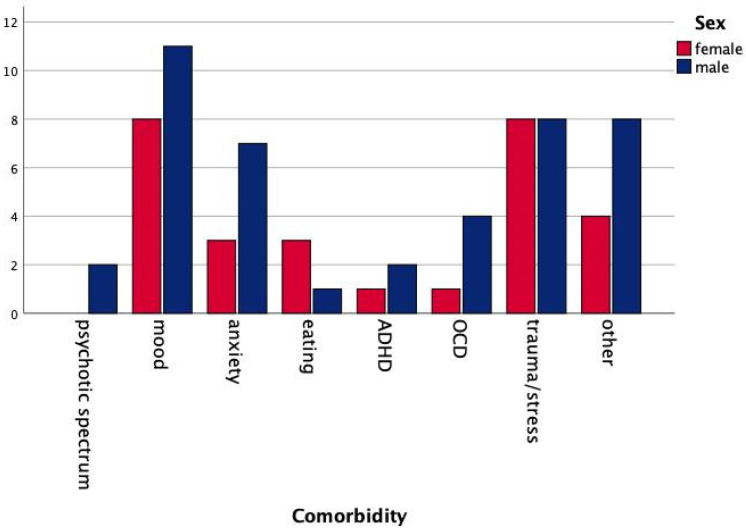
Frequency of comorbid mental disorders among males and females.

Sixty-three adult patients were consecutively referred to the adult outpatient service for the treatment of psychiatric disorders in adults with ASD in the 12-month period. Two subjects were excluded as a history of language/intellectual delay emerged during the first assessment. The final samples included sixty-one subjects (22 females (36.1%), 39 males (63.9%), age 28.5 ± 10.8 years) with a principal diagnosis of ASD with no language/intellectual deficit. While more detailed information about language skills and IQ scores was available for a subgroup of subjects, this is not included because of the great heterogeneity in method and age at the time of the assessment. Seven subjects (11.5%) did not report any comorbid diagnosis, 30 (49.2%) reported one comorbid diagnosis, and 24 subjects (39.3%) also received a second comorbidity diagnosis. The overall comorbidity was as follows: Two subjects (3.3%) were diagnosed with a psychotic spectrum disorder, 19 (31.1%) with a mood disorder, 10 (16.4%) with an anxiety disorder, four (6.6%) with an eating disorder, three (4.9%) with ADHD, five (8.2%) with OCD, 16 (26.2%) with a trauma and stress/related disorder, and 12 (19.7%) with other clinical problems (mainly sleep disorders or behavioral issues). Since the sample size was not sufficiently large, a statistical evaluation of sex differences in comorbid diagnoses was not performed. However, Figure 1 provides an at-a-glance overview of differences between males and females. The mean age at first referral to mental health services was 15.1 ± 13.9 years. The background history revealed that most subjects (*n* = 46, 75.4%) did not receive an ASD diagnosis at first evaluation by a mental health service. However, most of them (*n* = 46, 75.4%) received a treatment based on different diagnoses. An average time of eight years (8.1 ± 8.3) elapsed before the ASD diagnosis was made.

### 3.2. Sex Differences

The demographical and clinical characteristics of the study sample are shown in Table 1. Compared to males, females showed both a significantly greater delay in referral to mental health services and a significantly higher age at diagnosis of ASD. We then aimed to test whether different factors may relate to a greater diagnostic delay between males and females. To this end, we ran correlation analyses between AQ and AdAS Spectrum scores and, respectively, the age of ASD diagnosis and the time lapsing between first contact and ASD diagnosis, separately among women and men. Among men, a diagnostic delay inversely correlated with scores in the AdAS Spectrum total (r = −0.515, *p* = 0.01), Verbal communication (r = −0.479, *p* = 0.05), empathy (r = −0.425, *p* = 0.05) and Inflexibility and adherence to routine (r = −0.438, *p* = 0.01) domains, while no significant correlations were found with the age at the time of ASD diagnosis. Among women, a diagnostic delay positively correlated with the Attention to detail (r = 0.759, *p* = 0.05) scores, while the age at diagnosis of ASD positively correlated with the AdAS Spectrum Verbal communication (r = 0.551, *p* = 0.05) and Restricted interests and rumination (r = 0.535, *p* = 0.05) domain scores. While looking at the history of ASD diagnosis, three different situations were defined: 1. The diagnosis of ASD was correctly made at first evaluation; 2. ASD diagnosis was missed at first evaluation; 3. ASD was misdiagnosed with other mental disorders. As displayed in Table 1, females were less likely to be correctly diagnosed and more likely to be misdiagnosed at first evaluation than men.

**Figure 2:**
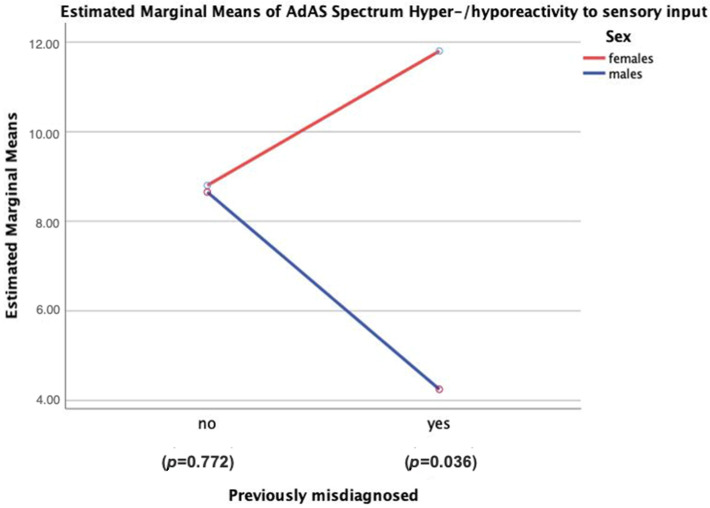
Gender effect on the Hyper/hyporeactivity to sensory input domain of the AdAS Spectrum in patients with or without previous misdiagnosis.

Notably, out of 10 females who were misdiagnosed, eight received a diagnosis of personality disorder, one of anxiety disorder and one of psychotic spectrum disorder. Among seven men who were misdiagnosed, four were labeled with ADHD, two with psychotic spectrum disorders and one with other behavioral issues. To investigate whether clinical phenotypes might relate differently to the chance of being misdiagnosed among women and men respectively, we ran a series of eleven factorial ANOVAs, using AdAS Spectrum and AQ domain scores as dependent variables and sex and previous misdiagnosis as independent ones. The results showed a significant effect of sex * misdiagnosis on the Restricted interests/rumination and Hyper/Hyporeactivity to sensory input domain scores of the AdAS Spectrum. While further comparing the two domain scores between males and females separately among subjects with or without previous misdiagnosis, females reported significantly higher scores than men in the Hyper/Hyporeactivity to sensory input domain, but only among subjects who were misdiagnosed (11.8 ± 4.8 vs. 4.3 ± 3.5, T = 2.596, sig. = 0.036) (Figure 2).

## 4. Discussion

The study was aimed to provide information about autism spectrum symptoms among males and females referred to a center for the treatment of psychiatric comorbidities in adults with ASD and to evaluate whether the path to ASD recognition in this patient group was differently characterized between men and women in terms of diagnostic delay, misdiagnosis and association with specific symptom domains. Consistent with extant knowledge about gender differences in ASD diagnosis, we found that both the age at first contact with mental health services and the age at the moment of ASD diagnosis were significantly higher in females compared to males (an average of a ten-year lag for both). One credited explanation for this datum is that male phenotypes are especially evident when compared to the normative behavior observable among neurotypical peers, while females’ problems are often less obvious. For example, some studies reported greater social and communicative deficits and greater repetitive behaviors among ASD males compared to females, [35] but other studies also pointed out that even when the level of impairment was similar, women tended to show interests, behaviors, and communicative features that were socially more acceptable and in line with gender stereotypes, increasing females’ chances of mimetizing typically developing subjects and conversely decreasing the probability of them being diagnosed and treated in a timely way [20]. It has to be noted, however, that despite females being referred and diagnosed later than males, the time-lag between the first evaluation and diagnosis was not different between sexes. One hypothesis is that widespread unfamiliarity with autism might increase the diagnostic delay in both sexes and smooth out differences. On the other hand, our sample was recruited in a service aimed to treat comorbid mental disorders in the course of ASD with no language or intellectual deficit, which might also explain the enhanced diagnostic delay, due to a more complex clinical picture prevailing over sex differences. Interestingly, different symptom dimensions were found to be significantly associated with diagnostic delay in women and men. In the latter group, scores obtained in the Verbal communication, Empathy and Inflexibility and adherence to routine domains of the AdAS Spectrum inversely correlated with a time-lag between the first evaluation and diagnosis, meaning that the lower the impairment they showed in these dimensions, the longer the time they waited to be diagnosed. Conversely, women reported a direct, strong correlation between diagnostic delay and the Attention to detail scores of the AQ questionnaire, meaning that a higher impairment in this area was associated with a greater diagnostic delay. Moreover, women’s age at diagnosis of ASD positively correlated with the AdAS Spectrum Verbal communication and Restricted interests and rumination scores. While they are apparently counterintuitive, these results may be explained in light of the frequent misdiagnosis affecting females in the autism spectrum. It seems possible, for instance, that girls with greater attention to details are initially diagnosed with other disorders, such as obsessive-compulsive personality or eating disorders, that share a similar cognitive profile [36,37]. Similarly, the positive correlation between the age at ASD diagnosis and impairments in verbal communication and interests may indicate that the way these impairments manifest in women do not fit classic knowledge of ASD and are therefore likely to be subsumed into other mental disorders. The inverse correlation of impaired verbal communication with diagnostic delay in men and its direct correlation with the age of diagnosis in women are especially noteworthy, since they suggest that ASD diagnosis is facilitated by verbal communication impairment among males and hindered among females. This might reflect the assumption that ASD is mainly prevalent among males, leading clinicians to easily refer verbal impairment to ASD in males and to other conditions—such as social anxiety [38]—in females. Consistent with this interpretation, while looking at patients’ clinical history, we found that males were significantly more likely than females to be diagnosed with ASD, while females were more likely than males to be misdiagnosed at the time of the first evaluation by mental health services. Despite the small sample size preventing us from further inferential analyses, previous misdiagnoses among females were mainly in the group of personality disorders. The distinction between ASD and personality disorders is very challenging indeed, especially in the case of mild and high-functioning autism and when the onset of the personality disorder is precocious [20]. Interestingly, women with a history of misdiagnosis showed higher scores in the AdAS Spectrum domain exploring sensory reactivity, a dimension involved in a tightly interwoven network with emotional dysregulation, borderline personality and ASD, [39,40,41] and only recently included among ASD symptom criteria. Subjective sensory overload has been shown to be more pronounced among adult females compared to males [42] and to relate to the risk of self-injuring in ASD populations [43]. Conversely, self-harm behavior is also a hallmark of borderline personality disorders, which may lead to the hypothesis of a mediating role of self-injury between heightened sensory reactivity and the risk of borderline misdiagnosis in females.

Our findings need to be considered in the light of obvious limitation. First, the small sample size and the small percentage of females can be greatly misleading from a statistical standpoint. Second, the study sample, including patients referred to a tertiary service for the treatment of comorbidities during the course of ASD with no language or intellectual delay, is representative of a limited population and does not allow a broad generalizability. Third, part of the data collection relied on patients’ and relatives’ reports. Despite nonsubjective sources of information being used as often as possible, we cannot rule out that recall biases affected our findings. It is possible that the lack of an accurate assessment of language skills and IQ scores as well as the lack of a measure of functioning hampered a broader perspective for the interpretation of our data. Despite the above limitations, our findings provide some gender-specific information about ASD patients seeking help for comorbid conditions and might be a primary ground for future research.

## Figures and Tables

**Table 1:** Socio-demographic and clinical characteristics of the study sample.

	Females *n* = 22	Males *n* = 39	t	Sig.
Age	30.2 (13.0)	27.5 (9.4)	4.799	0.409
Years of education	12.1 (3.3)	13.3 (2.8)	1.801	0.166
Age of first referral	21.1 (14.7)	11.7 (11.2)	2.398	**0.007**
Age at ASD diagnosis	29.4 (13.0)	19.8 (12.6)	0.431	**0.007**
Years between first referral and ASD diagnosis	8.2 (5.3)	8.0 (9.7)	4.721	0.889
AQ scores				
Total	32 (8.4)	29.3 (7.1)	0.084	0.294
Social skills	5.8 (2.7)	6.7 (2.5)	0.625	0.318
Attention to details	6.0 (2.6)	5.0 (2.5)	2.948	0.252
Attention switching	7.7 (1.8)	7.3 (1.4)	0.001	0.506
Communication	6.9 (2.3)	5.9 (2.3)	0.047	0.185
Imagination	4.7 (2.2)	5.3 (1.6)	1.315	0.364
AdAS Spectrum scores				
Total	92.9 (32.4)	89.7 (23.8)	3.722	0.721
Infancy/adolescence	12.6 (4.8)	12.8 (4.1)	1.020	0.889
Verbal comm	10.9 (4.2)	10.9 (4.2)	0.486	0.823
Nonverbal comm	15.1 (5.7)	14.1 (5.6)	0.220	0.570
Empathy	7.1 (3.0)	7.0 (2.7)	1.218	0.977
Adherence to routine/Inflexibility	23.3 (10.4)	23.0 (6.9)	4.781	0.941
Restricted interests/rumination	14.2 (5.5)	14.5 (4.9)	0.627	0.849
Hyper/Hyporeactivity to sensory input	9.8 (4.4)	7.7 (4.4)	0.002	0.149
			Chi-square	
Living with parents *n* (%)	12 (60)	25 (73.5)	0.538	0.463
Occupied *n* (%)	15 (68.2)	25 (64.1)	0.104	0.747
Previous vs. current diagnosis				
ASD diagnosis missed *n* (%)	12 (54.5)	20 (52.6)	0.021	0.886
Misdiagnosis of ASD *n* (%)	10 (45.5)	7 (17.9)	5.294	**0.021**
Previous treatment				
Any treatment *n* (%)	18 (81.8)	28 (73.7)	0.515	0.473
Previous antidepressants *n* (%)	13 (59.1)	21 (55.3)	0.083	0.773
Previous benzodiazepines *n* (%)	7 (31.8)	7 (18.4)	1.398	0.237
Previous Antipsychotics *n* (%)	6 (27.3)	18 (47.4)	2.344	0.126
Other previous treatments *n* (%)	4 (18.2)	6 (15.8)	0.057	0.811
Psychotherapy *n* (%)	14 (63.6)	23 (60.5)	0.057	0.811

Data are expressed as mean (SD) unless otherwise specified. Bold values denote statistical significance at the *p* < 0.05 level.

## Data Availability

The data presented in this study are available on request from the corresponding author.
